# Biting Midges of the Genus *Culicoides* in South Carolina Zoos

**DOI:** 10.1673/031.010.5501

**Published:** 2010-06-06

**Authors:** Mark P. Nelder, Dustin A. Swanson, Peter H. Adler, William L. Grogan

**Affiliations:** ^1^ 114 Long Hall, Department of Entomology, Soils & Plant Sciences, Clemson University, Clemson, South Carolina 29634; ^2^ Florida State Collection of Arthropods, Florida Department of Agriculture and Consumer Services, Gainesville, Florida 32608; ^3^ Current address: Enteric, Zoonotic, and Vector-Borne Disease Unit, Ministry of Health and Long-Term Care, 1075 Bay Street, Ontario, M5S 2B1, Canada

**Keywords:** biting flies, blood feeding, exotic animals, light traps, vectors

## Abstract

Biting midges of the genus *Culicoides* (Diptera: Ceratopogonidae) were collected during the summer of 2007 at the Greenville and Riverbanks Zoos in South Carolina with Centers for Disease Control and Prevention (CDC) traps equipped with ultraviolet or incandescent lights and baited with carbon dioxide. Sixteen species of *Culicoides* were collected, four of which represented more than 80%. They were *Culicoides guttipennis* (Coquillett), *Culicoides mulrenanni* Beck, *Culicoides obsoletus* (Meigen), and *Culicoides sanguisuga* (Coquillett). *C. guttipennis* was found on a dead colobus monkey and a dead golden-headed lion tamarin; *Culicoides husseyi* Wirth & Blanton was collected from an unidentified, abandoned bird's nest. Ultraviolet light-equipped traps captured significantly more *Culicoides* specimens than traps with incandescent light. Half of the collected species previously have been associated with vertebrate pathogens, indicating a potential risk to captive animals.

## Introduction

Zoos can provide a valuable surveillance system for a variety of zoonoses. The initial isolation and identification of West Nile virus in the United States, for example, was performed by employees of the Bronx Zoo, New York, NY, in 1999 (Centers for Disease Control and Prevention 1999). This initial isolation and recognition of West Nile virus became the impetus for a mosquito-surveillance program in the Bronx Zoo. However, most other American zoos have not initiated similar surveys to detect potential epizootics (Neider, unpublished). Eastern equine encephalitis virus was detected in whooping cranes, *Grus americana*, from the Patuxent Wildlife Research Center, Laurel, Maryland (Dein et al. 1986). The etiological agent of tularemia, *Francisella tularensis*, was detected in a squirrel monkey, *Saimiri* sp. (ca. 1989) and another unidentified monkey species (ca. 1988) in the Sacramento Zoo, California (Farlow et al. 2001). Epizootics in zoos, therefore, offer excellent opportunities to study the epidemiology of diseases with the ultimate goal of preventing further epizootics and epidemics.

While the presence of arthropods such as mosquitoes and cockroaches has been documented in zoological settings (Greenberg and Sanati 1970), biting midges have been overlooked, though there have been reports of the pathogens transmitted by these flies. For example, viral pathogens of birds (*Avipoxvirus* spp.), transmitted by biting midges and other biting flies, have been reported from two species of captive owls in two facilities in Florida (Deem et al. 1997). Species of *Haemoproteus*, avian blood parasites transmitted by biting midges and other biting flies, have been recorded from infected birds in zoos of California (Schrenzel et al. 2003; Ziman et al. 2004), Michigan (DeJong and Muzzall 2000), Oklahoma (Halpern and Bennett 1983), and Texas (Ferrell et al. 2007). These cases demonstrate that zoos can be foci of ceratopogonid-borne zoonoses, indicating the need for collaboration between veterinarians and entomologists.

To protect endangered animals and those in conservation and breeding programs, potential threats posed by biting midges should be evaluated. The objectives of this research were to survey biting midges and assess CDC traps equipped with ultraviolet or incandescent lights for their capture efficiency in two zoos in South Carolina: the Greenville Zoo in Greenville and the Riverbanks Zoo in Columbia.

## Materials and Methods

### Study sites

The Greenville Zoo (N 34° 50.58′ W 82° 23.24′; ∼294 MASL) is situated in the Western Division of the Piedmont on 4.5 hectares bordered by the Reedy River (5–10 m wide), a 1 m-wide unnamed stream, businesses, and residential areas. The Riverbanks Zoo and Botanical Gardens (N 34° 00.58′ W 81° 04.56′; ∼51 MASL) occupies 69 hectares in Columbia near the confluence of the Saluda and Broad Rivers. It is in the Sandhills Ecoregion on the Fall Line of the extreme western edge of the Atlantic Coastal Plain and is bordered by Interstate 126, business properties, and recreational areas.

### Collection of biting midges

Biting midges (*Culicoides* spp. (Diptera: Ceratopogonidae)) were collected with two types of traps: CDC traps with incandescent light (4-Watt, ABC trap, Clarke Mosquito Control, www.clarkemosquito.com) and CDC traps with ultraviolet light (Trap Model 1212, John W. Hock Company, www.johnwhock.com). All traps were suspended from trees at 1.5–2.0 m above ground in forests with approximately 50% canopy cover dominated by oaks, pines, and bamboo *Phyllostachys aurea* Carr. ex A. & C. Rivière (Cyperales: Poaceae). Ground cover consisted of woody plants such as poison ivy *Toxicodendron radicans* L. (Sapindales: Anacardiaceae) and Virginia creeper *Parthenocissus quinquefolia* L. (Vitales: Fitaceae). To provide a source of CO2, all traps were baited with 2.0–2.5 kg of dry ice in insulated dry-ice containers placed immediately above the traps (Igloo, Trap Model 812, John W. Hock Company). All traps were powered by 6-V, 12-amps per hour, rechargeable batteries (Model PS-6100F1, Power Sonic®, www.power-sonic.com).

Biting midges were collected from May to August 2007, using incandescent and UV traps. Two traps of each type were used in the Greenville Zoo, and three of each type were used in the Riverbanks Zoo. Incandescent and UV traps were run simultaneously for four consecutive nights per month, beginning at 16:00 h, and trap-collection containers (containing live biting midges) were retrieved at 08:00 h each of the four consecutive mornings. A trap night was defined as the period from deployment of a single trap until collection the next morning. Dead animals and nests, when available, also were examined for flies.

All specimens were fixed in 95% ethanol for subsequent clearing in phenol-alcohol and slide mounted in phenol-balsam, following the methods of Wirth and Marston (1968). *Culicoides* species were identified with keys and illustrations by Battle and Turner (1971), Blanton and Wirth (1979), and Wirth et al. (1985) and by comparison with specimens in the collection of WL Grogan. Representative specimens were deposited in the Clemson University Arthropod Collection and the synoptic collection of ceratopogonids maintained by WLG at Salisbury University.

### Statistical analyses

The mean numbers of biting midges per trap night were analyzed using a two-way ANOVA, comparing means between trap types, using month as a block, and following the general statistical methodology of Zar (1996). Tests were considered significant at p <0.05.

## Results

Sixteen species of *Culicoides* (Diptera: Ceratopogonidae) were collected from the zoos, with 10 species from the Greenville Zoo and 12 species from the Riverbanks Zoo (Table 1). Six species were present at both zoos: *Culicoides guttipennis* (Coquillett), *C. obsoletus* (Meigen), *C. paraensis* (Goeldi), *C. sanguisuga* (Coquillett), *C. scanloni* Wirth and Hubert, and *C. stellifer* (Coquillett). A total of 101 biting midges were collected at the Greenville Zoo, 90% of which were represented by two species, *C. guttipennis* (*n* = 59) and *C. obsoletus* (*n* = 32). At the Riverbanks Zoo, 88 specimens of *Culicoides* were captured, 81% of which belonged to four species: *C. sanguisuga* (*n* = 31), *C. mulrenanni* Beck (*n* = 25), *C. stellifer* (*n* = 8), and *C. guttipennis* (*n* = 7).

The incandescent traps, for both zoos combined, collected 38 individuals, representing 20% of the total number of *Culicoides* trapped. In the Greenville Zoo, the mean number of *C. guttipennis* per trap night was significantly higher in UV traps than in incandescent traps (F_2, 45_ = 12.8, p = 0.001) (Table 2). In the Riverbanks Zoo, the mean number of *C. mulrenanni* per trap night was not significantly higher in UV traps than in incandescent traps (F_2, 45_ = 3.7, p = 0.06). Mean numbers of *C. obsoletus* and *C. sanguisuga* caught per trap night were not analyzed statistically because they were not collected in incandescent traps.

*Culicoides* species were not observed feeding in the zoo, but females of *C. guttipennis* were collected from a dead colobus monkey, *Colobus guereza* Rüppell (Haplorrhini: Cercopithecidae), (one of two animals examined) and a dead golden-headed lion tamarin, *Leontopithecus chrysomelas* (Kuhl) (Haplorrhini: Cebidae), (one of three animals examined) in the Greenville Zoo. *C. husseyi* Wirth & Blanton was collected from one of four unidentified, abandoned bird nests in the Greenville Zoo.

**Table 1.**
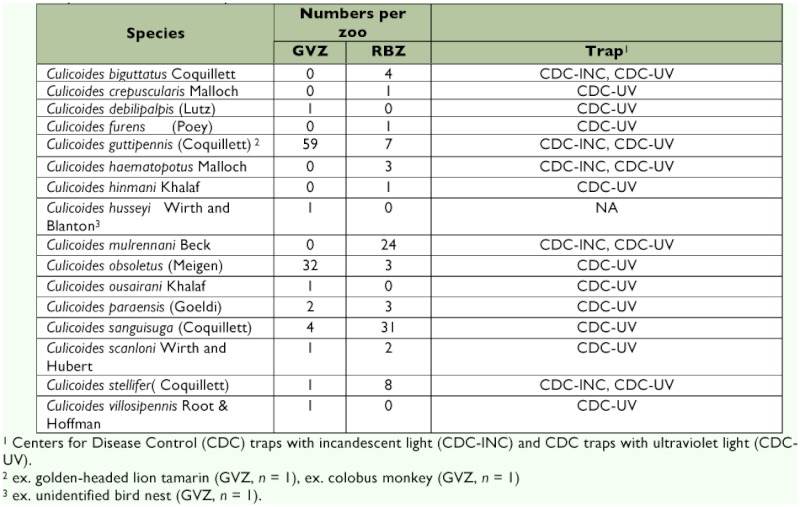
Biting midges collected from the Greenville Zoo (GVZ) and the Riverbanks Zoo (RBZ), South Carolina, with notes on the traps used to collect each species.

## Discussion

The 16 species of *Culicoides* collected in both zoos were typical of those in more rural areas of South Carolina. Suitable habitats for the immature stages of *Culicoides* are often abundant in zoos. The relatively higher numbers of *C. obsoletus* and *C. sanguisuga* in this study are likely due to the abundance of larval habitats of these two species in the zoos, including moist leaves and dung-laden straw (Jamnback 1965). *C. guttipennis* inhabits water-filled treeholes, a habitat found throughout the zoos and surrounding properties.

The collection of the coastal, salt marsh species, *Culicoides furens* (Poey), with a UV trap in the Riverbanks Zoo was unexpected. Adults of this species in the US Virgin Islands might be carried by prevailing winds for at least 6.4 km over low mountains (366 m) (Williams 1962), and adults in Panama could be carried up to 3.2 km by winds (Breeland and Smith 1962).

To our knowledge, *C. furens* has never been collected as far inland as central South Carolina; it possibly reached this locale by following the Congaree River inland from coastal habitats. The salt marsh mosquito *Ochlerotatus taeniorhynchus* Wiedemann (Diptera: Culicidae) is routinely collected in the vicinity of the Riverbanks Zoo (Neider, unpublished data).

Several species of *Culicoides* in this study have been associated with vertebrate pathogens (Table 3). For example, *C. stellifer* harbors Bluetongue virus in Alabama (Mullen and Anderson 1998) and West Nile virus in Louisiana (Sabio et al. 2006). Several species of *Culicoides* (e.g., *C. crepuscularis, C. haematopotus*, and *C. hinmani*) are vectors of *Haemoproteus* spp. and filarial parasites. Some species also might transmit nematodes, protozoans, and viruses to captive animals in zoos. *C. obsoletus* is a major vector of Bluetongue virus-8 in Europe and is the cause of epidemics throughout northern Europe (Mehlhorn et al. 2007). However, in the United States and Canada, *C. sonorensis* Wirth & Jones is the primary vector of Bluetongue virus (Borkent 2005), and *C. obsoletus* does not appear to be an effective vector in the Nearctic Region. Several other pathogens, if introduced into the United States, could possibly be transmitted by native species of *Culicoides*. For example, *C. paraensis* was collected from both zoos and is the only known vector of Oropouche virus, which causes nonfatal, febrile, flu-like infections in humans in South America and has been isolated from other primates and sloths (Mullen 2002).

**Table 2.**
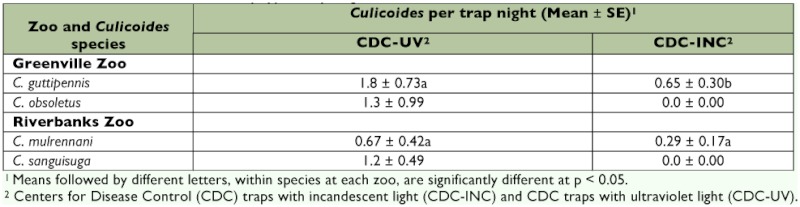
Mean number of *Culicoides* per trap night for the two most commonly collected species at the Greenville and Riverbanks Zoos, South Carolina, for two CDC-trap types, May-August 2007.

In zoos of South Carolina, surveillance for biting midges would best be served using UV traps because of the relatively greater abundance of biting midges caught by these traps and because no species was collected exclusively in incandescent traps. Competition from other incandescent light sources in and around a zoo likely reduces the collection efficiency of incandescent traps. The efficiency of traps for capturing biting midges differed geographically, and surveillance measures might need to be tailored for specific zoos. Surveillance programs should take into account the height at which biting midges seek hosts in the canopy. Some species in our study, such as *C. crepuscularis* Malloch, *C. haematopotus* Malloch, *C. hinmani* Khalaf, *C. scanloni*, and *C. villosipennis* Root & Hoffman, more frequently are collected higher in the canopy (Swanson 2007). Surveillance programs at zoos should consider the target species and vary the height of traps to sample the regional biting midge community.

**Table 3.**
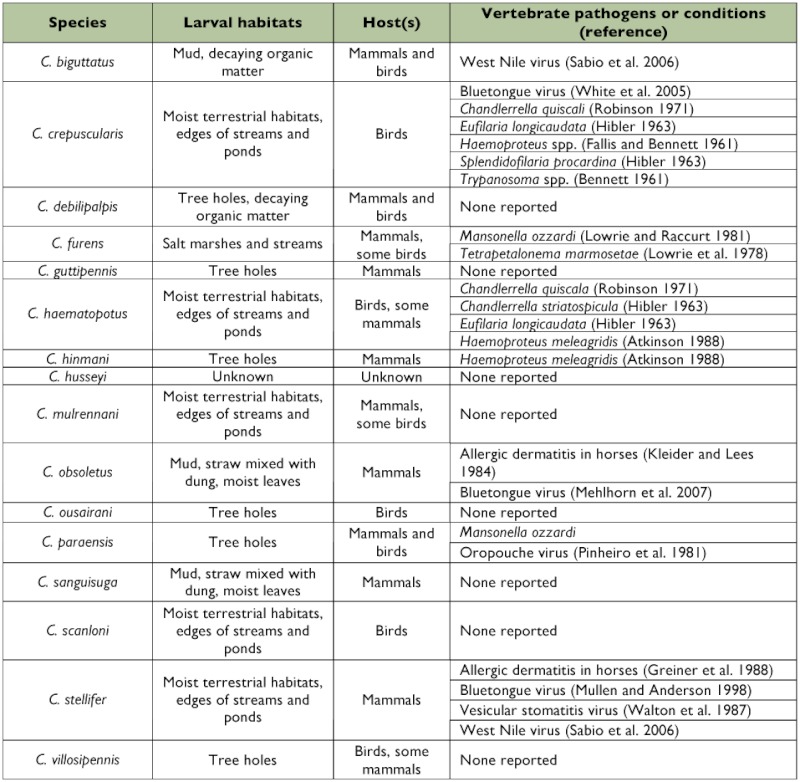
Larval habitats, host-feeding records, and pathogens associated with biting midges (*Culicoides*) collected at the Greenville and Riverbanks Zoos, South Carolina.

## References

[bibr01] Atkinson CT (1988). Epizootiology of *Haemoproteus meleagridis* (Protozoa: Haemosporina) in Florida: Potential vectors and prevalence in naturally infected *Culicoides* (Diptera: Ceratopogonidae).. *Journal of Medical Entomology*.

[bibr02] Battle FV, Turner C (1971). A systematic review of the genus *Culicoides* with a geographic catalog of the species occurring in the eastern United States north of Florida. *Insects of Virginia*, No. 3.

[bibr03] Bennett GF (1961). On the specificity and transmission of some avian trypanosomes.. *Canadian Journal of Zoology*.

[bibr04] Blanton FS, Wirth WW (1979). The sandflies (*Culicoides*) of Florida (Diptera: Ceratopogonidae). *Arthropods of Florida and Neighboring Land Areas*, Volume 10..

[bibr05] Borkent A, Marquardt WC (2005). Ceratopogonidae.. *Biology of Disease Vectors*.

[bibr06] Breeland SG, Smith JP (1962). Observations on the importance of flight range in the control of *Culicoides* in the Panama Canal Zone.. *Mosquito News*.

[bibr07] Centers for Disease Control and Prevention. (1999). Outbreak of West Nile-like viral encephalitis - New York, 1999.. *Morbidity and Mortality Weekly Report*.

[bibr08] Deem SL, Heard DJ, Fox JH (1997). Avian pox in eastern screech owls and barred owls from Florida.. *Journal of Wildlife Diseases*.

[bibr09] Dein FJ, Carpenter JW, Clark GG, Montali RJ, Crobbs CL, Tsai TF, Docherty DE (1986). Mortality of captive whooping cranes caused by eastern equine encephalitis virus.. *Journal of the American Veterinary Medical Association*.

[bibr10] DeJong RJ, Muzzall PM (2000). Hematozoa of waterfowl from the Kellogg Biological Station area in southwestern Michigan.. *Journal of Wildlife Diseases*.

[bibr11] Fallis AM, Bennett GF (1961). Ceratopogonidae as intermediate hosts for *Haemoproteus* and other parasites.. *Mosquito News*.

[bibr12] Farlow J, Smith KL, Wong J, Abrams M, Lytle M, Keim P (2001). *Francisella tularensis* strain typing using multiple-locus, variablenumber tandem repeat analysis.. *Journal of Clinical Microbiology*.

[bibr13] Ferrell ST, Snowden K, Marlar AB, Garner M, Lung NP (2007). Fatal hemoprotozoal infections in multiple avian species in a zoological park.. *Journal of Zoo and Wildlife Medicine*.

[bibr14] Greenberg B, Sanati M (1970). Enteropathogenic types of *Escherichia coli* from primates and cockroaches in a zoo.. *Journal of Medical Entomology*.

[bibr15] Greiner EC, Fadok VA, Rabin EB (1988). Equine *Culicoides* hypersensitivity in Florida: Biting midges collected in light traps near horses.. *Medical and Veterinary Entomology*.

[bibr16] Halpern N, Bennett GF (1983). *Haemoproteus* and *Leucocytozoon* in birds of the Oklahoma City Zoo.. *Journal of Wildlife Diseases*.

[bibr17] Hibler CP (1963). *Onchocercidae (Nematoda: Filarioidea) of the American Magpie, Pica pica hudsonia (Sabine), in Northern Colorado.*.

[bibr18] Jamnback H (1965). The *Culicoides* of New York State (Diptera: Ceratopogonidae).. *New York State Museum and Science Service Bulletin*.

[bibr19] Kleider N, Lees MJ (1984). *Culicoides* hypersensitivity in the horse: 15 cases in southwestern British Columbia.. *Canadian Veterinary Journal*.

[bibr20] Lowrie RC, Eberhard ML, Orihel TC (1978). Development of *Tetrapetalonema marmosetae* to the infective stage in *Culicoides hollensis* and *C. furens*.. *Journal of Parasitology*.

[bibr21] Lowrie RC, Raccurt C (1981). *Mansonella ozzardi* in Haiti: II. Arthropod vector studies.. *American Journal of Tropical Medicine and Hygiene*.

[bibr22] Mehlhorn H, Walldorf V, Klimpel S, Jahn S, Jaeger F, Eschweiler J, Hoffmann B, Beer M (2007). First occurrence of *Culicoides* ooso/eto-transmitted bluetongue virus epidemic in Central Europe.. *Parasitology Research*.

[bibr23] Mullen GR, Mullen G, Durden L (2002). Biting midges (Ceratopogonidae).. *Medical and Veterinary Entomology*..

[bibr24] Mullen GR, Anderson RR (1998). Transmission of orbiviruses by *Culicoides* Latreille species (Ceratopogonidae) among cattle and white-tailed deer in the southeastern United States, Abstracts Volume.

[bibr25] Pinheiro FP, Travassos da Rosa APA, Travassos da Rosa JFS, Ishak R, Freitas RB, Gomes MLC, LeDuc JW, Oliva OFP (1981). Oropouche virus: I. A review of clinical, epidemiological, and ecological findings.. *American Journal of Tropical Medicine and Hygiene*.

[bibr26] Robinson EJ (1971). *Culicoides crepuscularis* (Malloch) (Diptera: Ceratopogonidae) as a host for *Chandlerella quiscali* (von Linstow, 1904) comb. n. (Filarioidea: Onchocercidae).. *Journal of Parasitology*.

[bibr27] Sabio IJ, Mackay AJ, Roy A, Foil LD (2006). Detection of West Nile virus RNA in pools of three species of ceratopogonids (Diptera: Ceratopogonidae) collected in Louisiana.. *Journal of Medical Entomology*.

[bibr28] Schrenzel SD, Maalouf GA, Keener LL, Gaffney PM (2003). Molecular characterization of malarial parasites in captive passerine birds.. *Journal of Parasitology*.

[bibr29] Swanson DA (2007). *Vertical distribution of hematophagous flies (Diptera) and their avian hematozoan parasites in South Carolina forests.*.

[bibr30] Walton TE, Webb PA, Kramer WL, Smith GC, Davis T, Holbrook FR, Moore CG, Schiefer TJ, Jones RH, Janney GC (1987). Epizootic vesicular stomatitis in Colorado, 1982: Epidemiologic and entomologie studies.. *American Journal of Tropical Medicine and Hygiene*.

[bibr31] White DM, Wilson WC, Blair CD, Beaty BJ (2005). Studies on overwintering of bluetongue viruses in insects.. *Journal of General Virology*.

[bibr32] Williams RW (1962). Observations on the bionomics of Ceratopogonidae.. *Mosquito News*.

[bibr33] Wirth WW, Dyce AL, Peterson BV (1985). An atlas of wing photographs, with a summary of the numerical characters of the Nearctic species of *Culicoides* (Diptera: Ceratopogonidae).. *Contributions of the American Entomological Institute* (Gainesville, Florida).

[bibr34] Wirth WW, Marston N (1968). A method for mounting small insects on microscope slides in Canada Balsam.. *Annals of the Entomological Society of America*.

[bibr35] Zar JH (1996). *Biostatistical Analysis*.

[bibr36] Ziman M, Colagross-Schouten A, Griffey S, Stedman B (2004). *Haemoproteus* spp. and *Leucocytozoon* spp. in a captive raptor population.. *Journal of Wildlife Diseases*.

